# Important methodological flaws in the recently published clinical prediction model the REMEMBER score

**DOI:** 10.1186/s13054-019-2363-3

**Published:** 2019-03-07

**Authors:** Anders Granholm, Anders Perner, Aksel Karl Georg Jensen, Morten Hylander Møller

**Affiliations:** 1 Department of Intensive Care 4131, Copenhagen University Hospital – Rigshospitalet, Inge Lehmanns Vej 5, 2100 Copenhagen, Denmark; 2 Centre for Research in Intensive Care, Blegdamsvej 9, 2100 Copenhagen, Denmark; 3 Section of Biostatistics, University of Copenhagen, Øster Farimagsgade 5, 1014 Copenhagen, Denmark

We have with interest read the recently published paper by Wang et al. in *Critical Care*, which proposes a new clinical prediction model—the REMEMBER score—to predict in-hospital mortality in patients undergoing venoarterial extracorporeal membrane oxygenation (VA-ECMO) after coronary artery bypass grafting [1]. The topic is clinically relevant; however, the study suffers from important methodological shortcomings, which hamper the validity of the findings and conclusions presented.

First, the study is critically underpowered. An effective sample size (minimum number of events or non-events) of ≥ 10 per candidate variable is recommended [2], and often more are required [3]. This number is 4–5 for the REMEMBER score (74 non-events/17 candidate variables). Consequently, the risk of random errors, overfitting, and inflated performance estimates is high. The performance will almost certainly deteriorate when used in other populations, and the single-centre design increases this risk.

Second, calibration was only assessed using the Hosmer-Lemeshow *Ĉ* test, which is highly sensitive to sample size and unable to indicate lack of fit when power is low [2]. Lack of significance for this test does not equal adequate fit, and calibration plots or regressions of the predicted versus observed outcomes are recommended [2].

Third, comparing a newly developed prediction model with existing models using the development dataset is not recommended [2], as this approach is biassed towards favouring the new model, especially when the risk of overfitting is high. A different cohort independent of model development must be used [2].

Additional important limitations include the long recruitment period where standards of care may have changed, the use of a non-fixed-time mortality outcome affected by discharge practices, the lack of external validation, and the reporting, which lacks information on sample size considerations and missing data handling, and inadequately acknowledges important limitations [4].

We are worried about the consequences if the REMEMBER score is used to select patients for VA-ECMO as suggested [1]. VA-ECMO is a costly and highly invasive treatment with severe potential adverse effects, and we agree that research within this area is highly needed [5]. Prediction models are relevant in this context; however, it is paramount that such models are developed, validated, and reported appropriately [2, 4]. If not, their use may put patients at risk and lead to inappropriate use of resources. Developing and validating a trustworthy clinical prediction model in this very selected patient population likely requires international, multicentre collaboration [5].

## Abbreviations

VA-ECMO: Venoarterial extracorporeal membrane oxygenation
