# Supplementary material for: Important methodological flaws in the recently published clinical prediction model the REMEMBER score
Source: Crit Care. 2019 Mar 7;23:71. doi: 10.1186/s13054-019-2363-3 (PMC6407253; doi:10.1186/s13054-019-2363-3)
Supplement: Related texts

# Reply: Authors’ response

**Authors:** Liangshan Wang, Hong Wang, Xiaotong Hou

We are grateful to Granholm et al. for their interesting and valuable comments on our paper.

The REMEMBER score was developed according to the SAVE score [6], ENCOURAGE score [7], and two published recommendations [8, 9]. Actually, we have mentioned that the single-centre design and absence of external validation may limit the generalizability of the REMEMBER score. Theoretically, the number of patients included in this study might be a little limited regarding the number of variables included in the model, which is similar in the ENCOURAGE score. Thus, we performed a bootstrap analysis, and it showed similar results, confirming the stability of the original model. In addition, a post hoc random forest analysis [10] was performed, in which the six pre-ECMO parameters of the REMEMBER score were the top 10 risk factors. As for calibration, we also investigated the relationship between the predicted and observed outcomes grouped by REMEMBER score classes using a method that was similar to the calibration plots and found that there was a very good overlap between observed and expected mortality in all four groups. Calibration regression of the predicted versus observed outcomes is always recommended for external validation. Over the last 14 years, our surgical approach to CABG and standards of care have not changed much over the last 14 years, and year of ECMO was not associated with mortality by univariable analysis (*p* = 0.698). Given the absence of external validation, we have moderated the conclusion and did not use confirmatory terms in the paper. Prospective studies are needed to externally validate the scoring system before it can be widely applied.
